# A commentary on ‘The impact of diabetes on postoperative outcomes following spine surgery: a meta-analysis of 40 cohort studies with 2.9 million participants’

**DOI:** 10.1097/JS9.0000000000000801

**Published:** 2023-10-04

**Authors:** Sung Huang Laurent Tsai

**Affiliations:** a Department of Orthopaedic Surgery, Chang Gung Memorial Hospital, Keelung Branch, Keelung; b School of Medicine, Chang Gung University, Taoyuan, Taiwan

*Dear Editor,*

We read with interest the paper recently published in the *International Journal of Surgery* titled ‘The impact of diabetes on postoperative outcomes following spine surgery: a meta-analysis of 40 cohort studies with 2.9 million participants’.

In this study, Luo *et al*.^1^ present a significant contribution to the understanding of the association between diabetes and postoperative complications in the context of spinal surgery. The meta-analysis, comprising 40 cohort studies and an impressive participant pool of 2.9 million individuals, meticulously examines the relationship between diabetes and postoperative outcomes in the context of spine surgery. The study employs a systematic approach to collect, evaluate, and synthesize data from a wide range of sources, thereby enhancing its overall robustness.

First and foremost, the study effectively highlights the increased risk of postoperative complications associated with diabetes, including postoperative infection, reoperation, surgery-related mortality, and blood transfusion. These findings are in line with previous research and underscore the clinical significance of diabetes management in the preoperative period^2–4^. One notable strength of the study is its comprehensive approach, incorporating a large dataset and utilizing a thorough search strategy across multiple databases. The adherence to PRISMA (Preferred Reporting Items for Systematic Reviews and Meta-Analyses) guidelines and the use of the Newcastle–Ottawa Scale for quality assessment enhance the study’s credibility. The employment of various statistical techniques, such as sensitivity analysis and subgroup analysis, is also commendable, as it assesses the robustness of results and addresses potential sources of bias. However, the study could benefit from further exploration of certain critical aspects. One notable gap in the data extraction process is the omission of information regarding diabetes medications. Diabetes management, including medication regimens, can profoundly impact postoperative outcomes^5^. The type, dosage, and duration of diabetes medications are crucial factors that could influence the risk of postoperative complications^6^. Considering that diabetes management plays a critical role in surgical outcomes, the absence of data on diabetes medication in the data extraction process represents a significant limitation. Future studies and meta-analyses in this field should consider incorporating information about diabetes medication as a critical variable to provide a more comprehensive understanding of the impact of diabetes on postoperative complications.

Second, the lack of consistency in reporting how diabetes was measured is a notable limitation in this meta-analysis. Understanding how diabetes was diagnosed and measured in the included studies is crucial for interpreting the results and drawing meaningful conclusions. However, Table 1 reveals that many of the studies did not provide clear information regarding their criteria for diagnosing and measuring diabetes^7^. The inconsistency in reporting how diabetes was diagnosed and measured across the included studies is a limitation. Different criteria for diabetes diagnosis may capture different subsets of patients with varying degrees of glycemic control. This can impact the clinical relevance of the findings, as patients with well-controlled diabetes may have different postoperative outcomes compared to those with poorly controlled diabetes^8^. Future research should prioritize standardized criteria and transparent reporting to enhance the reliability and applicability of findings in clinical practice.

Third, while the subgroup analysis is valuable, it’s important to emphasize the need for similar subgroup analyses on other major potential effect modifiers. Factors such as the severity of diabetes, medication use, HbA1c levels, and the number of spine levels fused/decompressed are known to influence postoperative outcomes in patients with diabetes^9–11^. Conducting subgroup analyses on these variables would contribute to a more comprehensive understanding of the nuances within this patient population and their impact on surgical outcomes. Preoperative glycemic control, as reflected by HbA1c levels, is a critical factor in reducing the risk of postoperative complications, including infections. Studies have shown that achieving target HbA1c levels before surgery can significantly improve outcomes^10^. Therefore, it is essential to discuss and include analyses related to the optimal level of HbA1c that should be controlled before spine surgery.

These additional subgroup analyses could help identify specific patient profiles that are at higher or lower risk for postoperative complications, allowing healthcare providers to tailor interventions and management strategies accordingly. Furthermore, it could shed light on whether certain factors, such as stringent glycemic control or medication adjustments, are particularly beneficial in reducing complications in specific patient subgroups. Incorporating these analyses into future research efforts would not only enhance the depth of evidence but also provide clinicians with more targeted guidance for managing patients with diabetes undergoing spinal surgery. It would be a valuable extension of the current study’s efforts to explore the complex relationship between diabetes and postoperative outcomes.

The study appropriately highlights the clinical implications of its findings, emphasizing the importance of early evaluation of blood glucose levels and effective intervention. Expanding on these implications and addressing potential challenges in implementing recommendations could further guide healthcare providers in improving patient care. A forward-looking statement on potential avenues for future research in this area, such as large prospective cohort studies, would add depth to the discussion.

In conclusion, the study is a commendable effort to shed light on the impact of diabetes on postoperative outcomes in spinal surgery. By addressing the mentioned issues and exploring further subgroup analyses, future research can build upon this foundation and provide even more valuable insights for clinical practice, ultimately benefiting patients undergoing spinal surgery with diabetes.

## Ethical approval

Not applicable.

## Consent

Not applicable.

## Sources of funding

Not applicable.

## Author contribution

All authors have approved the manuscript and agree with the submission.

## Conflicts of interest disclosure

There are no conflicts of interest.

## Research registration unique identifying number (UIN)

Not applicable.

## Guarantor

Sung Huang Laurent Tsai.

## Data availability statement

Not applicable.

## Provenance and peer review

Not commissioned, externally peer-reviewed.
